# Genome-Wide Identification and Expression Analysis of the *WOX* Gene Family in Pepino (*Solanum muricatum*), Followed by Cloning and Subcellular Localization of *SmWOX5* and *SmWOX15*

**DOI:** 10.3390/cimb48080782

**Published:** 2026-07-31

**Authors:** Xuebing Zhu, Yunhe Cao, Xuemei Sun, Shipeng Yang, Lihui Wang

**Affiliations:** 1 Academy of Agriculture and Forestry Sciences, Qinghai University, Xining 810016, China; 15297190798@163.com (X.Z.); yunhe.cao@origene.cn (Y.C.); sunxuemei1008@163.com (X.S.); 2 Laboratory for Research and Utilization of Germplasm Resources in Qinghai Tibet Plateau, Qinghai University, Xining 810016, China

**Keywords:** *WOX* gene family, pepino (*Solanum muricatum*), tissue-specific expression, subcellular localization, adventitious root formation

## Abstract

WOX transcription factors play conserved roles in plant adventitious root development, but the *WOX* family in pepino (*Solanum muricatum*) has not been systematically characterized. To investigate this family, genome-wide identification and expression analysis were performed. A total of 15 *SmWOX* genes were identified, unevenly distributed across 12 chromosomes and phylogenetically divided into three clades: WUS (11), Intermediate (3), and Ancient (1). RT-qPCR revealed that most *SmWOX* genes showed the highest expression in apical buds and the lowest in leaves; among them, *SmWOX5* and *SmWOX15* exhibited significantly higher expression in roots. These two genes were subsequently cloned and subjected to subcellular localization analysis. *SmWOX5* (492 bp, 163 amino acids) and *SmWOX15* (2514 bp, 837 amino acids) were both predicted as non-transmembrane, non-secretory proteins, with differences in phosphorylation site distribution and structural conformations. Subcellular localization showed that *SmWOX5* is exclusively nuclear, whereas *SmWOX15* is predominantly nuclear with partial plasma membrane distribution. The *WOX* family in pepino exhibits evolutionary conservation with signs of functional divergence. The high root expression and differential localization of *SmWOX5* and *SmWOX15* suggest their distinct regulatory roles in root development and adventitious root formation, providing candidate genes for improving cutting propagation efficiency in pepino (*Solanum muricatum*).

## 1. Introduction

Pepino (*Solanum muricatum*, 2*n* = 24) is a perennial herbaceous species belonging to the Solanaceae family and native to the Andes Mountains of South America [1]. The fruit is nutrient-dense and holds significant economic value, coupled with strong developmental prospects [2,3]. In commercial production, pepino is propagated predominantly through asexual means, specifically stem cuttings, a method valued for its high survival rate and reliable preservation of varietal characteristics [4,5]. Given that the key to successful cutting propagation lies in the initiation and development of adventitious roots, and that pepino stem nodes possess a highly developed capacity for adventitious root formation, exploring the molecular regulatory mechanisms underlying root development holds significant application value for optimizing cutting propagation techniques and improving seedling quality.

*WOX* (WUSCHEL-related homeobox) is a plant-specific transcription factor belonging to the homeobox superfamily [6,7,8]. Its protein harbors a homeodomain comprising 60–66 highly conserved amino acid residues, which mediates sequence-specific binding to target gene DNA via a helix-turn-helix (HTH) motif [9,10]. *WOX* transcription factors play a central regulatory role in key developmental processes in plants, including embryogenesis, stem cell maintenance, and organogenesis [11,12,13]. Recently, the evolutionarily conserved function of the *WOX* family in regulating adventitious root formation has been demonstrated across a broad spectrum of plant species [14,15]. In Arabidopsis, *WOX11* and *WOX12* are activated by wound-induced auxin signaling, thereby promoting the fate transition of procambial cells into root founder cells and initiating the adventitious root developmental program [16]. Similarly, *OsWOX11* in rice and *PeWOX11a/b* in poplar are implicated in adventitious root formation [17,18]. Moreover, *MdWOX4b* in apple and *RhWOX331* in rose have likewise been shown to promote adventitious and lateral root development [19,20]. These findings demonstrate that *WOX* transcription factors play a conserved and essential role in adventitious root development across plant species, acting through the integration of hormonal signals, regulation of cell fate specification, and promotion of root primordium formation. Despite this, no systematic, genome-wide identification of the *WOX* gene family has been conducted in pepino, and the molecular mechanisms governing root development in this crop remain poorly understood. Consequently, such knowledge gaps impede genetic improvement efforts.

In this study, we conducted a genome-wide identification of the *WOX* gene family in pepino, followed by phylogenetic analysis and tissue-specific expression profiling. Particular attention was focused on *SmWOX5* and *SmWOX15*, two root-enriched genes that were cloned, subjected to bioinformatic analyses, and validated for subcellular localization via transient expression in tobacco. Collectively, these findings provide a molecular foundation for understanding adventitious root regulation and offer candidate genes for improving cutting propagation efficiency in pepino.

## 2. Materials and Methods

### 2.1. Plant Materials

The pepino cultivar ‘Qingtianxiang’ (*Solanum muricatum*) used in this study was registered with the Qinghai Academy of Agriculture and Forestry Sciences. According to the official registration record, this cultivar is suitable for protected cultivation in the Hehuang Valley and the Qaidam Basin of Qinghai Province, as well as for open-field cultivation at altitudes between 1800 and 2200 m. The plant materials used in this study were obtained from the germplasm repository of the Academy and maintained under standard cultivation conditions. Plants were grown at the Horticultural Innovation Base of the Academy. Stem segments were harvested from uniformly developed plants exhibiting consistent lateral bud germination rates and were used for vegetative propagation via stem cuttings. Sixty days after cutting, four tissue types, apical buds, lateral buds, roots, and leaves, were collected. Root tissues were utilized for gene cloning, whereas all four tissue types were employed for RT-qPCR analysis.

### 2.2. Methods

#### 2.2.1. Identification of the *WOX* Gene Family in Pepino

The pepino reference genome assembly used in this study was generated by our research group [21,22]. The *WOX* protein sequences of *Arabidopsis thaliana* were retrieved from the Arabidopsis Information Resource (TAIR; https://www.arabidopsis.org/, accessed on 12 August 2024) as reference sequences. A proteome-wide BLASTP search was conducted against the predicted pepino protein dataset using TBtools (v2.0) (https://github.com/CJ-Chen/TBtools accessed on 23 August 2024) to identify candidate *WOX* proteins. Additionally, the Hidden Markov Model (HMM) profile for the WOX family homeodomain (PF00046) was downloaded from the Pfam database (http://pfam-legacy.xfam.org/, accessed on 19 August 2024), and HMMER (http://hmmer.org/, accessed on 21 August 2024) was employed to search against the same pepino protein dataset. The candidate genes were subsequently defined by the union of protein hits obtained from both BLASTP and HMMER analyses, and the corresponding gene IDs were then mapped to the pepino genome for systematic naming.

#### 2.2.2. Bioinformatic Analysis of the *SmWOX* Gene Family

*WOX* protein sequences from tomato, pepper, potato, and maize were retrieved from the PlantTFDB database (https://planttfdb.gao-lab.org/, accessed on 29 August 2024). Multiple sequence alignment was conducted using MAFFT (v7.490) (https://mafft.cbrc.jp/alignment/software/, accessed on 23 August 2024), and a maximum-likelihood (ML) phylogenetic tree was inferred with IQ-TREE (V2.2.0) (1000 bootstrap replicates). The resulting tree was visualized using iTOL (V6.9.1) (https://itol.embl.de/, accessed on 25 August 2024). Conserved motifs in the pepino *WOX* proteins were identified using MEME (v5.5.6) (http://meme-suite.org/, accessed on 28 August 2024), with the maximum number of motifs set to 12. Physicochemical properties including amino acid length, molecular weight, isoelectric point (pI), instability index, aliphatic index, and grand average of hydropathicity (GRAVY) were computed for each *SmWOX* protein using the ExPASy ProtParam tool (https://www.expasy.org/, accessed on 1 September 2024). Chromosomal locations of the *SmWOX* genes were determined based on the pepino genome annotation file using TBtools. Gene duplication events within the pepino genome, as well as interspecific syntenic relationships between pepino and other species, were analyzed using MCScanX. For each *SmWOX* gene, the 2000 bp genomic sequence upstream of the translation start codon (ATG) was extracted from the pepino reference genome. Putative cis-regulatory elements in these promoter regions were predicted using PlantCARE (http://bioinformatics.psb.ugent.be/webtools/plantcare/html/, accessed on 2 September 2024) and graphically represented using TBtools (v2.0).

#### 2.2.3. Expression Analysis of *SmWOX* Genes in Different Tissues

To investigate the expression patterns of *SmWOX* genes across different pepino tissues, total RNA was extracted from apical buds, lateral buds, roots, and leaves using the OMEGA HP Plant RNA Kit (OMEGA, Norcross, GA, USA). RNA integrity and concentration were assessed spectrophotometrically (A260/A280 and A260/A230 ratios) and by 1% agarose gel electrophoresis. High-quality RNA samples were reverse-transcribed into cDNA using the All In One 5× RT MasterMix (ABM, Richmond, BC, Canada). Quantitative real-time PCR (RT-qPCR) was performed with the FastReal qPCR PreMix (SYBR Green) (TIANGEN, Beijing, China), using UBQ119 as the internal reference gene; the PCR reaction system and cycling program are detailed in Figure S1A. Gene-specific primers were designed using Primer 5.0 (sequences listed in Table S1A). For each tissue type, three independent biological replicates were collected, and each biological sample was analyzed in technical triplicate. Relative gene expression levels were calculated using the 2^−ΔΔCT^ method. Data are presented as mean ± SD (*n* = 3 biological replicates). Statistical significance was determined by one-way ANOVA followed by Tukey’s HSD post hoc test for multiple comparisons, with *p* < 0.05 considered statistically significant.

#### 2.2.4. Cloning and Bioinformatics Analysis of *SmWOX5* and *SmWOX15*

To investigate the functional roles of *SmWOX5* and *SmWOX15* in adventitious root formation in pepino (*Solanum muricatum*), comprehensive gene structure and bioinformatic analyses were performed. Total RNA was extracted from root tissues of two-month-old stem cuttings, and first strand cDNA was synthesized as described in Section 2.2.3. Full-length coding sequences of *SmWOX5* and *SmWOX15* were amplified via PCR using PrimeSTAR^®^ Max DNA Polymerase (TIANGEN, Beijing, China) and gene-specific primers (Table S1B). The PCR reaction system and thermal cycling program are detailed in Figure S1B. Amplification products were resolved by 1% agarose gel electrophoresis. Target bands were excised, purified, and recovered using the TaKaRa MiniBEST Agarose Gel DNA Extraction Kit Ver. 4.0 (Takara Bio Inc., Kusatsu, Japan) and subsequently sequenced by Sangon Biotech Co., Ltd. (Shanghai, China). Protein structural features including transmembrane domains, signal peptides, phosphorylation sites, secondary structure, and tertiary structure were predicted using TMHMM-2.0 (https://services.healthtech.dtu.dk/services/TMHMM-2.0/, accessed on 23 August 2024), SignalP-6.0 (https://services.healthtech.dtu.dk/services/SignalP-6.0/, accessed on 24 August 2024), NetPhos 3.1 (https://services.healthtech.dtu.dk/services/NetPhos-3.1/, accessed on 25 August 2024), SOPMA (https://npsa-prabi.ibcp.fr/cgi-bin/npsa_automat.pl, accessed on 10 September 2024), and SWISS-MODEL (https://swissmodel.expasy.org/, accessed on 15 September 2024), respectively.

#### 2.2.5. Subcellular Localization of *SmWOX5* and *SmWOX15*

##### Vector Construction

To construct subcellular localization expression vectors, the full-length coding sequences of *SmWOX5* and *SmWOX15* excluding stop codons were amplified from pepino root cDNA using gene-specific primers bearing homologous overhangs (Table S1C). The pCAMBIA2300-GFP vector served as the backbone and was double-digested with SacI and XbaI restriction enzymes. Digested products were resolved by 1% agarose gel electrophoresis, and the linearized vector fragments were purified. Amplified target gene fragments were then cloned into the linearized vector via homologous recombination, following the manufacturer’s protocol for the NovoRec^®^ Plus One Step PCR Cloning Kit (Novoprotein, Suzhou, China). Following the recombination reaction, the ligation products were transformed into *Escherichia coli* DH5α competent cells. Transformants were selected on LB solid medium supplemented with kanamycin (50 mg/L). Individual kanamycin-resistant colonies were picked, and plasmids were isolated. The resulting recombinant plasmids were subsequently introduced into *Agrobacterium tumefaciens* GV3101 competent cells by heat shock. Transformed *Agrobacterium* cells were plated onto dual-antibiotic selective medium containing kanamycin and rifampicin. Positive clones were confirmed by colony PCR. The successfully assembled recombinant constructs were designated pCAMBIA2300-SmWOX5-GFP and pCAMBIA2300-SmWOX15-GFP.

##### Fluorescence Detection

Positive *Agrobacterium* clones were resuspended in MES buffer (10 mM MgCl_2_, 10 mM MES, pH 5.6, supplemented with 200 µM acetosyringone) to an OD_600_ of 0.6–0.8. The bacterial suspension was infiltrated into the abaxial surface of the third or fourth fully expanded leaves of 4–6-week-old *Nicotiana benthamiana* plants using a needleless syringe. For each construct, three independent infiltration experiments were performed, with three plants per replicate and two leaves infiltrated per plant. Following infiltration, plants were incubated at 22–25 °C under a 16 h light/8 h dark photoperiod for 48 h. Confocal laser scanning microscopy was performed using an FV1000-ASW microscope. GFP fluorescence was excited at 488 nm and detected through a 500–530 nm bandpass filter; chlorophyll autofluorescence was simultaneously collected using a 650–750 nm bandpass filter under the same 488 nm excitation. Nuclei were counterstained with DAPI (excitation: 358 nm; emission: 400–450 nm). Composite images were generated by merging GFP, chlorophyll (CHL), DAPI, and differential interference contrast (DIC) channels to determine the subcellular localization of the target proteins.

## 3. Results

### 3.1. Identification and Phylogenetic Analysis of SmWOX Family Members

**Figure 1:**
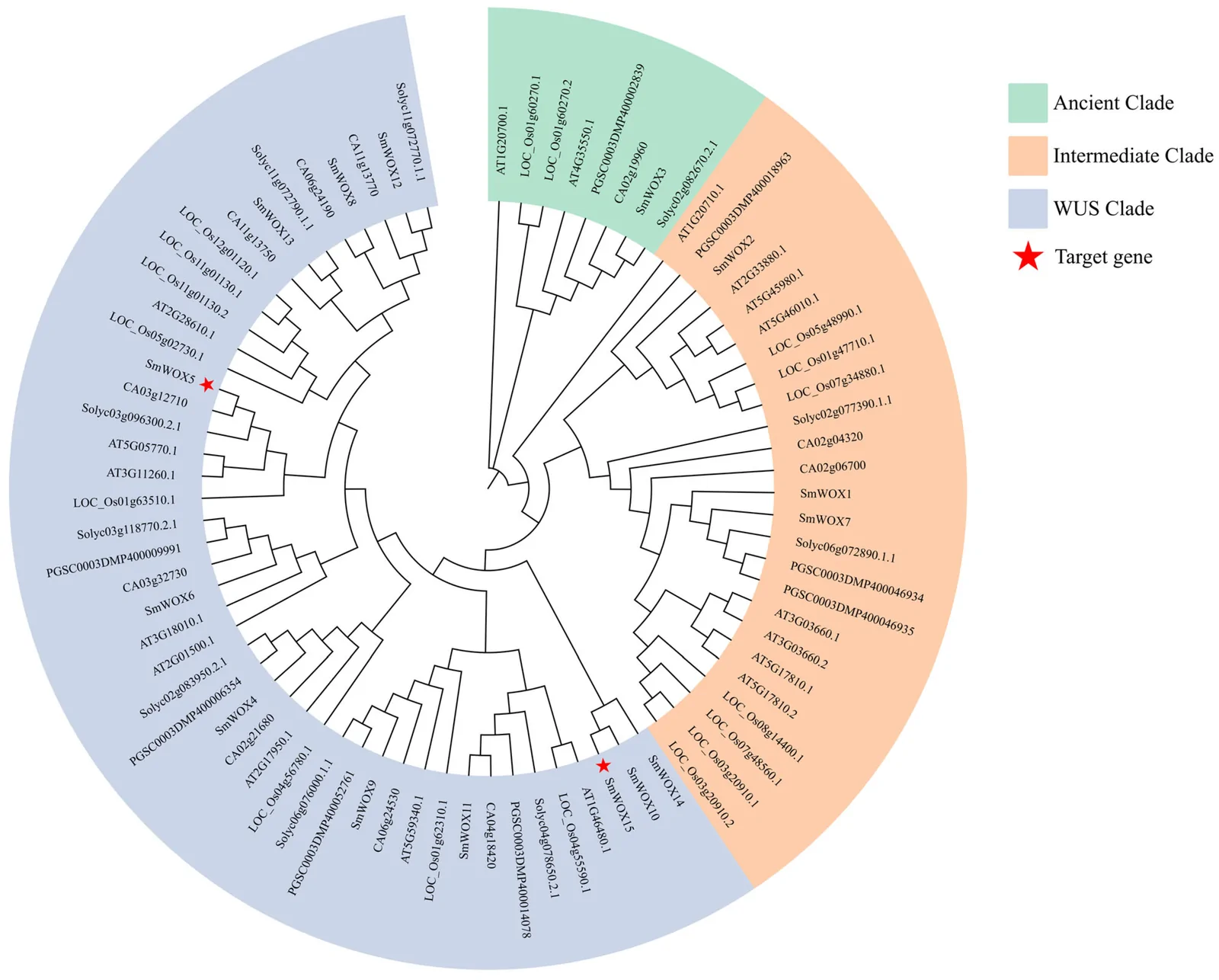
Phylogenetic tree of WOX proteins.

A total of 15 *WOX* family members were identified in the pepino (*Solanum muricatum*) genome and designated *SmWOX1*–*SmWOX15* according to their physical order along the chromosomes (Table 1). To elucidate their evolutionary relationships, the deduced amino acid sequences of the 15 *SmWOX* proteins were aligned with those of *WOX* proteins from *Arabidopsis thaliana* (18), tomato (*Solanum lycopersicum*, 10), pepper (*Capsicum annuum*, 11), rice (*Oryza sativa*, 17), and potato (*Solanum tuberosum*, 8). A maximum-likelihood phylogenetic tree was subsequently constructed (Figure 1). Phylogenetic analysis revealed that all *WOX* genes clustered into three well-supported clades: the WUS clade, the Intermediate clade, and the Ancient clade. These three clades are distinguished by their phylogenetic positions and conserved motifs, with the WUS clade characterized by the presence of the WUS-box motif, whereas the Ancient clade lacks this motif and represents the most evolutionarily conserved lineage. Among them, the WUS clade comprised the largest number of members, while the Ancient clade contained the fewest.

The pepino *WOX* family members were most abundantly represented in the WUS clade, comprising 11 members: *SmWOX4*, *SmWOX5*, *SmWOX6*, *SmWOX8*, *SmWOX9*, *SmWOX10*, *SmWOX11*, *SmWOX12*, *SmWOX13*, *SmWOX14*, and *SmWOX15*. Among these, *SmWOX4* is orthologous to Arabidopsis *AtWUS*, and *SmWOX5* is orthologous to *AtWOX5*, suggesting conserved functional roles in shoot apical meristem and root apical meristem development, respectively. *SmWOX1*, *SmWOX2*, and *SmWOX7* cluster within the intermediate clade, whereas *SmWOX3* belongs to the Ancient clade. Among the six species analyzed, Arabidopsis is the only one harboring multiple Ancient clade *WOX* genes; all other species possess a single representative. Phylogenetic analyses revealed that pepino exhibits higher sequence homology and closer evolutionary relationships with tomato, potato, and pepper, whereas it displays lower homology and more distant evolutionary divergence from Arabidopsis and rice.

### 3.2. Bioinformatics Analysis of SmWOX Gene Family

#### 3.2.1. Physicochemical Property Analysis

Prediction of the physicochemical properties of the pepino *WOX* family proteins revealed the following ranges: amino acid length, 163–898; molecular weight, 19,263.73–98,884.23 Da; isoelectric point (pI), 5.47–9.98; instability index, 40.93–69.53; and aliphatic index, 49.3–87.03. The grand average of hydropathicity (GRAVY) values ranged from 1.031 to 0.103 consistently negative, indicating that all *WOX* proteins are hydrophilic in nature (Table S2).

#### 3.2.2. Conserved Motif and Gene Structure Analyses

**Figure 2:**
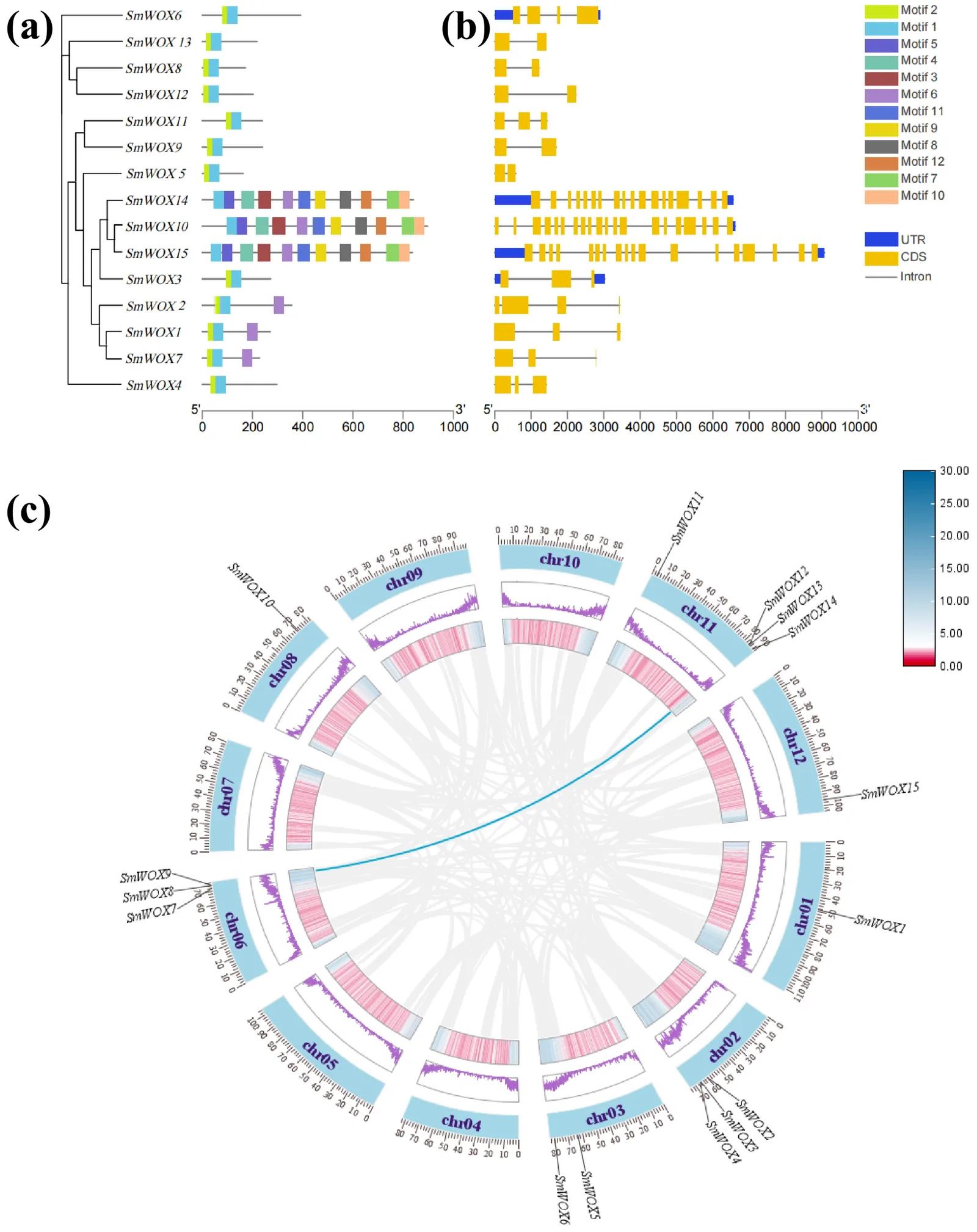
Conserved motifs, gene structures, and chromosomal distribution of the pepino WOX family. (**a**) Conserved motifs identified in pepino WOX proteins. (**b**) Gene structure (exon–intron organization) of pepino WOX genes. (**c**) Chromosomal distribution of pepino WOX genes on 12 chromosomes.

Conserved motif prediction analysis identified a total of 12 conserved motifs across the *WOX* family. Motif 1 was present in all family members, suggesting its potential role as a core functional motif essential for *WOX* protein activity. With the exception of *SmWOX10*, *SmWOX14*, and *SmWOX15*, which each harbored 11 conserved motifs, the remaining family members contained only 2–3 conserved motifs, all of which included Motif 2. In contrast, Motifs 3–5 and 7–12 were exclusively detected in *SmWOX10*, *SmWOX14*, and *SmWOX15* (Figure 2a).

Gene structure analysis showed that 10 of the 15 family members (*SmWOX13*, *SmWOX8*, *SmWOX12*, *SmWOX11*, *SmWOX9*, *SmWOX5*, *SmWOX2*, *SmWOX1*, *SmWOX7*, and *SmWOX4*) contained only CDS regions, while five members (*SmWOX6*, *SmWOX14*, *SmWOX10*, *SmWOX15*, and *SmWOX3*) contained UTR regions. Among them, *SmWOX10* had the highest number of CDS (20), *SmWOX14* and *SmWOX15* each contained 18 CDS, and the remaining members had 2–4 introns (Figure 2b).

#### 3.2.3. Chromosomal Localization and Duplication Event Analyses

The pepino *WOX* genes were unevenly distributed across the 12 chromosomes, with no *SmWOX* genes located on chromosomes 4, 5, 7, 9, or 10. Chromosome 11 harbored the highest number of *WOX* genes (four: *SmWOX11*, *SmWOX12*, *SmWOX13*, and *SmWOX14*). Chromosome 2 contained *SmWOX2*, *SmWOX3*, and *SmWOX4*; chromosome 6 contained *SmWOX7*, *SmWOX8*, and *SmWOX9*; chromosome 3 carried two genes (*SmWOX5* and *SmWOX6*); and chromosomes 1, 8, and 12 each harbored a single gene (*SmWOX1*, *SmWOX10*, and *SmWOX15*, respectively). Gene duplication analysis identified only one pair of segmentally duplicated genes among the pepino *WOX* family, *SmWOX8* and *SmWOX12* (Figure 2c).

#### 3.2.4. Promoter Cis-Regulatory Element Analysis

**Figure 3:**
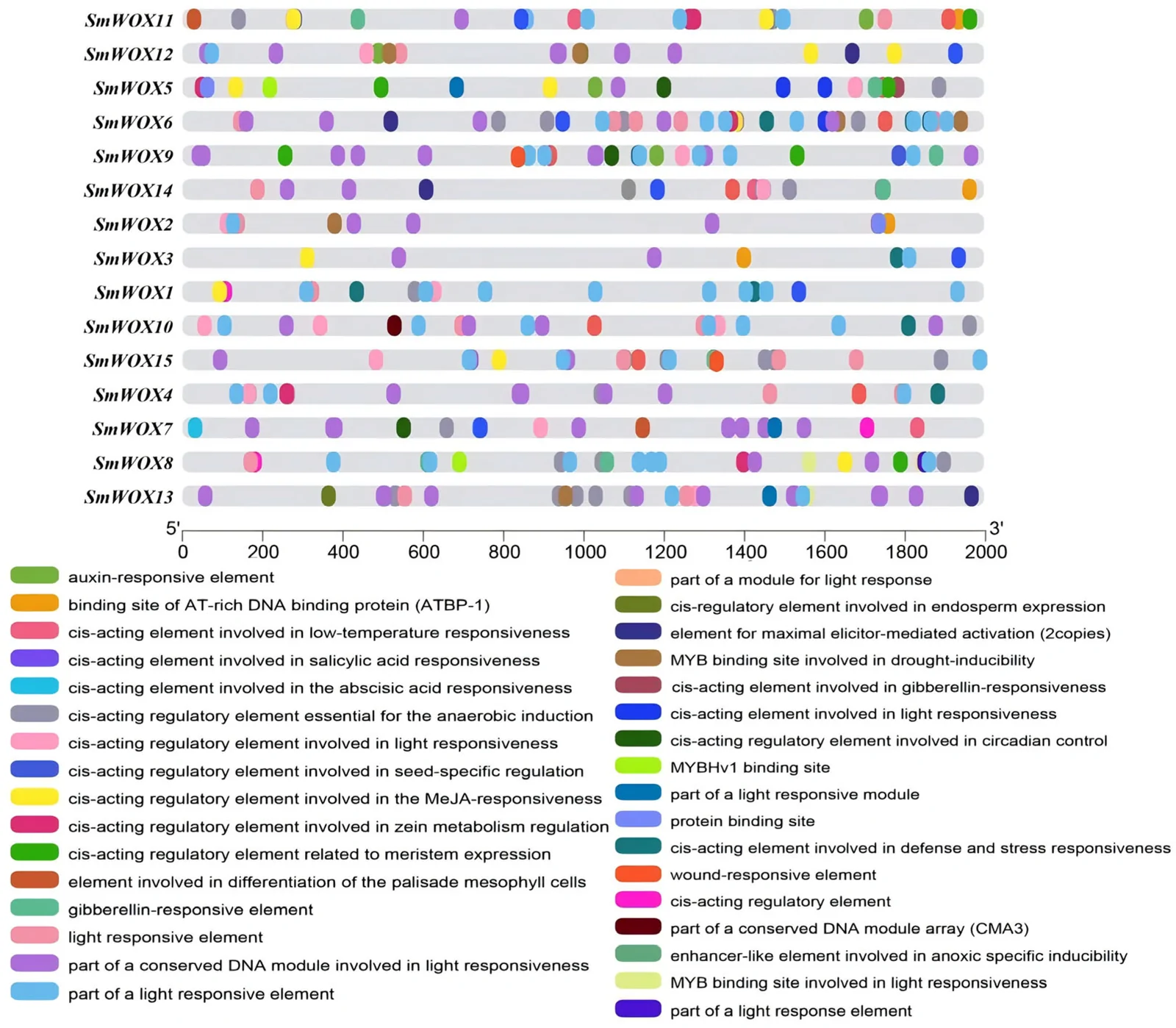
Cis-regulatory element analysis of *SmWOX* gene promoters.

To analyze the functional characteristics of the *SmWOX* genes, the 2000 bp genomic sequences upstream of the translational start codon (ATG) for each gene were retrieved for promoter analysis. Cis-regulatory elements were predicted using the PlantCARE database. The results revealed that, in addition to core promoter elements (e.g., TATA-box and CAAT-box), the identified cis-elements were predominantly grouped into four functional categories: light-responsive elements, phytohormone-responsive elements, abiotic stress-responsive elements, and elements associated with plant growth and development. Among these, light-responsive elements were the most abundant; ten distinct types were identified and were widely distributed across multiple *SmWOX* promoters. Seven abiotic stress-related elements were detected, including two anaerobic induction elements (ARE), one low-temperature responsive element (LTR), two MYB binding sites (MBS), and two wound responsive elements (WUN-motif). Furthermore, six phytohormone responsive elements and eight growth- and development-related elements were identified. With respect to phytohormone responsiveness, cis-elements conferring sensitivity to abscisic acid (ABA), auxin (IAA), gibberellin (GA), methyl jasmonate (MeJA), and salicylic acid (SA) were present in the promoters of most *SmWOX* genes. Regarding growth and development, the ATBP-1 motif represents an AT-rich sequence binding site, whereas CMA3 is implicated in the regulation of developmental stage transitions. Notably, several *SmWOX* genes also harbored cis-elements linked to meristem-specific expression, seed-specific regulation, palisade mesophyll cell differentiation, and endosperm expression (Figure 3). Collectively, these findings suggest that the *WOX* gene family plays pivotal roles in mediating light signaling and phytohormone-regulated processes during pepino development.

#### 3.2.5. Synteny Analysis Across Species

**Figure 4:**
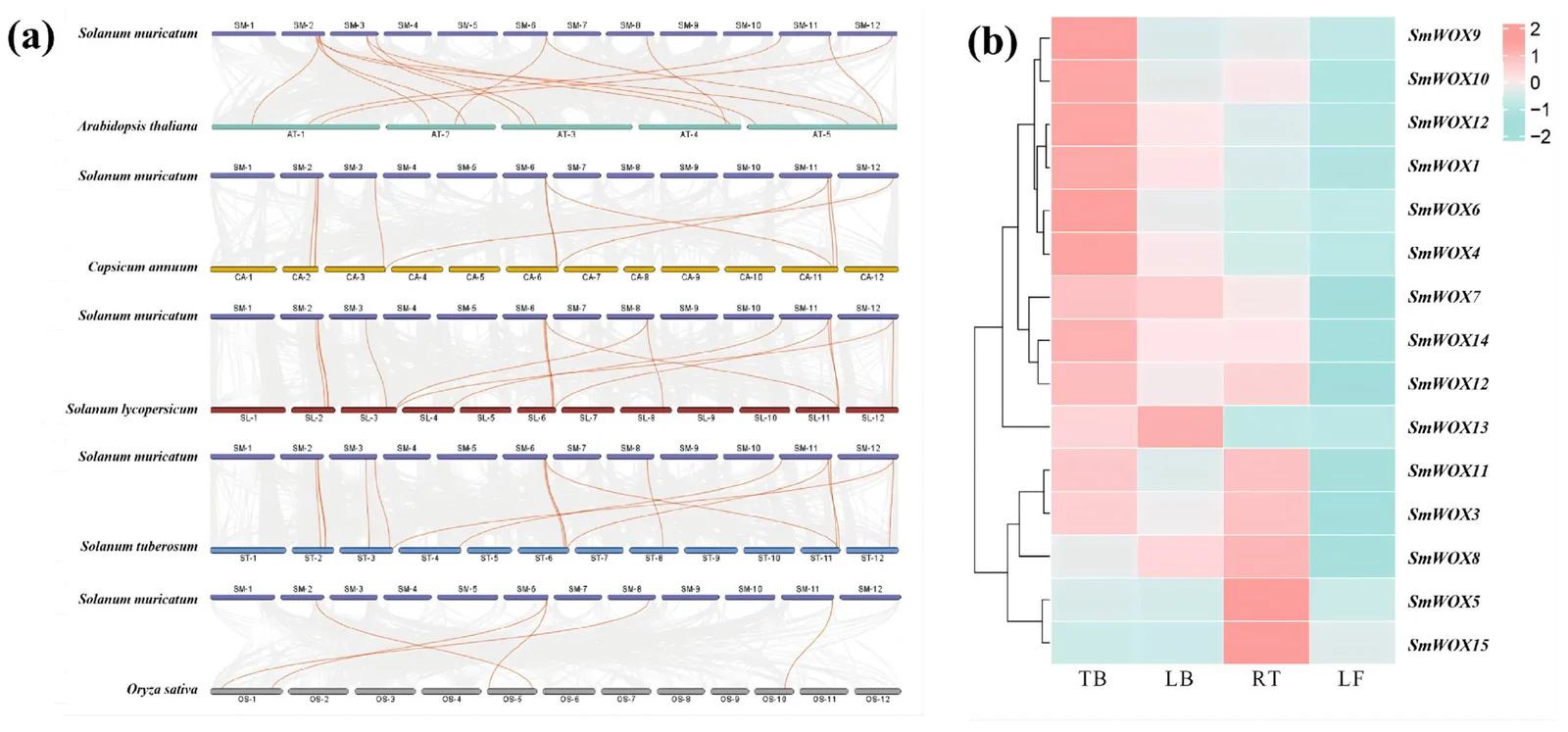
Chromosomal localization and tissue-specific expression patterns of *SmWOX* genes in pepino. (**a**) Chromosomal localization and synteny analysis of *SmWOX* genes. The 12 chromosomes are indicated by bars, and the scale bar represents megabases (Mb). Segmental duplication pairs are connected by red lines. (**b**) Tissue-specific expression patterns of *SmWOX* genes. Expression levels in apical bud, lateral bud, root, and leaf are shown using a color scale from 5 to 35, representing relative expression values. The UBI119 gene was used as the internal control.

Synteny analysis revealed variations in the number of *WOX* syntenic gene pairs between pepino and the other examined species. A total of 14 syntenic gene pairs were identified between pepino and *Arabidopsis thaliana*, located on pepino chromosomes 2, 3, 6, 8, 11, and 12. Between pepino and pepper (*Capsicum annuum*), 11 syntenic gene pairs were detected, distributed across chromosomes 2, 3, 6, 11, and 12. The highest numbers of syntenic gene pairs were observed between pepino and tomato (*Solanum lycopersicum*) and between pepino and potato (*Solanum tuberosum*), with 15 pairs in each case likely reflecting their close phylogenetic relationship as members of the Solanaceae family. In contrast, the fewest syntenic gene pairs were found between pepino and rice (*Oryza sativa*), with only five pairs distributed on chromosomes 2, 6, 8, and 11 consistent with their more distant evolutionary divergence. Notably, *SmWOX2*, *SmWOX8*, *SmWOX9*, and *SmWOX10* formed homologous gene pairs across all five species, suggesting that these genes may play critical roles in plant evolution (Figure 4a).

### 3.3. Differential Expression Analysis in Different Tissues

To further characterize the expression patterns and potential functional roles of the *WOX* gene family across different pepino (*Solanum muricatum*) tissues, RT-qPCR was employed to quantify the transcript levels of all 15 identified *WOX* genes in four representative tissues: apical buds, lateral buds, roots, and leaves. The results revealed that most *WOX* genes exhibited highest expression in apical buds, followed by lateral buds and roots, with the lowest expression consistently observed in leaves. Notably, apical and lateral buds constitute the primary sites of meristematic activity. Within these tissues, members of the WUS clade and all members of the Intermediate clade showed relatively higher expression levels, including *SmWOX9*, *SmWOX10*, *SmWOX2*, *SmWOX1*, *SmWOX6*, and *SmWOX4*, thereby reinforcing the central involvement of pepino *WOX* genes in the regulation of plant growth and development (Figure 4b). In contrast, *SmWOX5* and *SmWOX15* displayed moderate expression in roots; notably, *SmWOX15* expression was significantly higher than that of *SmWOX5* (Table S3), suggesting a potentially specialized role for *SmWOX15* in root development and root meristem maintenance.

### 3.4. Sequence and Structural Features of SmWOX5 and SmWOX15

#### 3.4.1. Gene Analysis of *SmWOX5* and *SmWOX15*

Agarose gel electrophoresis of the PCR amplicons yielded distinct bands corresponding to the expected sizes (Figure S2A). Sanger sequencing confirmed that the full length coding sequences of *SmWOX5* and *SmWOX15* are 492 bp and 2514 bp in length, respectively, encoding polypeptides of 163 and 837 amino acids.

#### 3.4.2. Bioinformatics Analysis of *SmWOX5* and *SmWOX15*

Transmembrane structure prediction indicated that neither *SmWOX5* nor *SmWOX15* harbors any transmembrane domains across their full length sequences, as evidenced by stable and consistently low prediction probability profiles. These results strongly suggest that both proteins are non-transmembrane, cytosolic or nuclear localized factors (Figure S3A). Signal peptide prediction analysis revealed that the N-terminal sequences of both proteins failed to meet the established threshold for signal peptide identification, indicating the absence of canonical signal peptides and supporting the conclusion that *SmWOX5* and *SmWOX15* are not classical secretory proteins (Figure S3B).

Protein phosphorylation site analysis identified multiple potential phosphorylation sites in both *SmWOX5* and *SmWOX15*; however, marked differences were observed in both the number and spatial distribution of these sites. *SmWOX5*, characterized by a relatively short polypeptide chain (~160 amino acids), possesses a limited number of predicted phosphorylation sites, predominantly localized on serine residues, followed by threonine and, to a lesser extent, tyrosine. Only a small subset of these sites exhibited phosphorylation propensity scores marginally exceeding the prediction threshold, implying that *SmWOX5* may undergo relatively constrained, context-specific phosphorylation-mediated regulation. In contrast, *SmWOX15*, featuring a substantially longer sequence (~800 amino acids), contains numerous predicted phosphorylation sites distributed broadly along its primary structure. Notably, sites with scores significantly above the threshold are enriched within the N-terminal region (residues 0–300), where serine and threonine residues display markedly elevated phosphorylation potential; several tyrosine residues in this region also exhibit clear, above-threshold phosphorylation propensity. Collectively, these findings suggest that *SmWOX15* is likely engaged in more extensive and dynamic signal perception, transduction, and protein–protein interaction networks (Figure S3C).

Secondary structure prediction indicated that *SmWOX5* is predominantly composed of random coils (66.26%), with α-helices (20.86%) and β-sheets (12.88%) constituting the remainder. Tertiary structure modeling further revealed a comparatively simple three-dimensional architecture, supported by a high template–model sequence identity (85.80%) and a Global Model Quality Estimation (GMQE) score of 0.6. For SmWOX15, the secondary structure exhibited a more complex and compact structural organization (Figure S3D).

### 3.5. Subcellular Distribution of SmWOX5 and SmWOX15

#### 3.5.1. Vector Construction

Detection of the recombinant vectors by 1% agarose gel electrophoresis revealed distinct bands corresponding to the expected sizes of pCAMBIA2300-SmWOX5-GFP and pCAMBIA2300-SmWOX15-GFP, confirming successful vector construction (Figure S2B).

#### 3.5.2. Subcellular Localization Results

**Figure 5:**
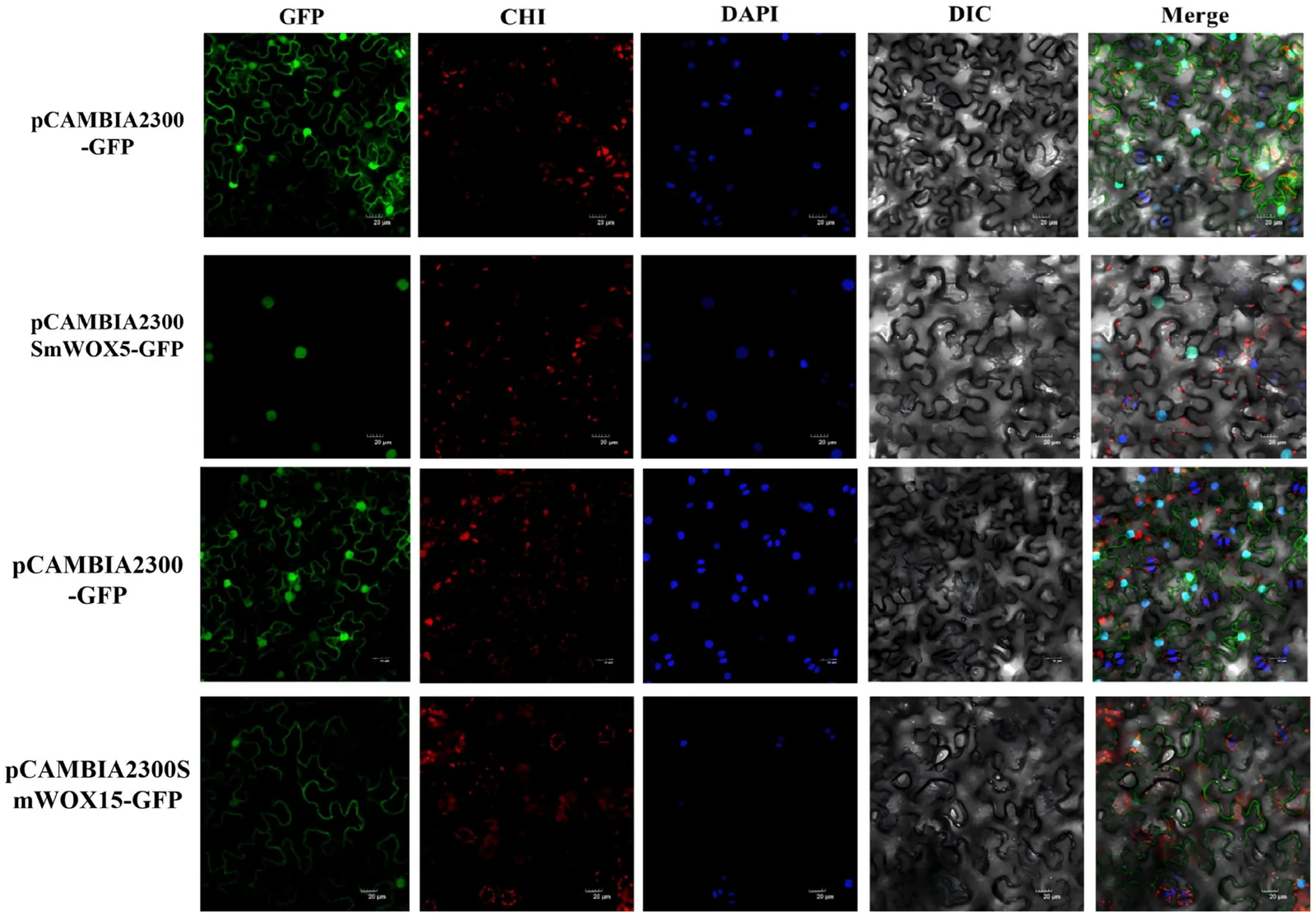
Subcellular localization of *SmWOX5* and *SmWOX15* proteins in tobacco leaf epidermal cells. The recombinant vectors pCAMBIA2300-SmWOX5-GFP and pCAMBIA2300-SmWOX15-GFP were transiently expressed in *Nicotiana benthamiana* leaves. GFP fluorescence (green), chlorophyll autofluorescence (red), and DAPI-stained nuclei (blue) were visualized using confocal microscopy. Merged images (Merge) and bright-field (DIC) images are shown. Bars = 20 μm. The empty vector (pCAMBIA2300-GFP) was used as a control.

Using the empty vector pCAMBIA2300 as a control, green fluorescence was observed throughout the cytoplasm and nucleus, consistent with the expected diffuse localization of free GFP. Subcellular localization analysis of the pCAMBIA2300-SmWOX5-GFP and pCAMBIA2300-SmWOX15-GFP fusion proteins revealed that GFP fluorescence signals were predominantly localized to the nucleus, with the GFP signals detected in the same regions as DAPI-stained nuclei. Notably, in addition to nuclear accumulation, SmWOX15-GFP also displayed distinct GFP fluorescence at the plasma membrane (Figure 5). These findings indicate that *SmWOX5* is exclusively nuclear, whereas *SmWOX15* exhibits predominant nuclear localization with partial association at the plasma membrane.

## 4. Discussion

### 4.1. Evolutionary Features of the WOX Gene Family in Pepino

In this study, a total of 15 *WOX* genes were identified in the pepino (*Solanum muricatum*) genome. This number is comparable to those found in other Solanaceae species, such as tomato (10), pepper (11), and potato (11), and is identical to that in Arabidopsis thaliana (15). In contrast, it is higher than in rice (14). These results suggest that, while the WOX family size is generally conserved in Solanaceae and close to that of Arabidopsis, a slight variation exists among different lineages [23,24]. Phylogenetic analysis classified these genes into three evolutionarily conserved clades, namely the WUS clade (11 members), the Intermediate clade (3 members), and the Ancient clade (1 member), a tripartite organization widely observed across angiosperms [14,25,26]. The WUS clade constituted the largest proportion (73.3%) of WOX genes in pepino, a distribution pattern consistent with those in tomato and potato, implying relatively active evolutionary diversification of this clade within the Solanaceae. However, notable differences exist in the WUS clade composition: pepino has 11 WUS-clade members, whereas tomato and potato have fewer (eight), suggesting that the WUS clade may have undergone slight expansion in pepino after divergence from other Solanaceae lineages. In contrast, the Ancient clade comprised only a single gene, *SmWOX3*, mirroring the pattern observed in most angiosperms and indicating strong purifying selection acting on this deeply conserved lineage [27]. Synteny analysis revealed that pepino shares the greatest number of syntenic gene pairs with tomato and potato (15 pairs each) and the fewest with rice (5 pairs), aligning with the well-established close phylogenetic relationship among the three Solanaceae species. Notably, only one pair of segmentally duplicated genes, *SmWOX8* and *SmWOX12*, was detected, suggesting that segmental duplication has not been a major driver of *WOX* family expansion in pepino [19]. These results suggest that while the overall *WOX* family structure is conserved in Solanaceae, species-specific variations in gene number and clade composition may reflect divergent evolutionary trajectories. Importantly, while previous WOX studies in tomato, pepper, and potato have been largely limited to bioinformatic predictions and expression profiling, our study goes beyond by providing direct experimental validation of subcellular localization for two root-enriched WOX proteins in a Solanaceae species.

### 4.2. High Root Expression of SmWOX5 and SmWOX15 and Its Relationship with Cutting Propagation

Tissue-specific expression analysis revealed that *SmWOX5* and *SmWOX15* exhibited significantly higher transcript levels in roots compared with other tissues, with *SmWOX15* showing markedly greater expression than *SmWOX5*. *WOX5* is a well-established core regulator of root apical meristem (RAM) maintenance, with evolutionarily conserved functions across diverse plant species, including Arabidopsis thaliana and Oryza sativa [28,29]. Thus, the pronounced root-specific expression of *SmWOX5* strongly suggests a conserved role in RAM maintenance in *Solanum muricatum*. The exceptionally high expression of *SmWOX15* in roots is particularly noteworthy, given that adventitious root formation is essential for successful stem-cutting propagation [30]. Accumulating evidence implicates multiple WOX family members in adventitious root development: *OsWOX11* regulates crown root initiation in rice [18,31], and *WOX* genes in Eucalyptus display dynamic, stage-specific expression patterns during adventitious root formation [32]. Since pepino is predominantly propagated via stem cuttings, the robust root-specific expression of *SmWOX15* implies a potential functional role in adventitious root initiation, particularly in response to wound-induced auxin signaling during cutting propagation. Consistent with this hypothesis, promoter sequence analysis revealed that most *SmWOX* genes harbor canonical auxin-responsive cis-elements (AuxREs), further supporting their regulation by auxin-mediated signaling pathways [33].

### 4.3. Differences in Subcellular Localization Between SmWOX5 and SmWOX15

Subcellular localization analysis revealed that *SmWOX5* is exclusively localized to the nucleus, whereas *SmWOX15* exhibits predominant nuclear localization with partial accumulation at the plasma membrane. The strictly nuclear localization of *SmWOX5* is consistent with its predicted role in root apical meristem (RAM) maintenance and aligns with the canonical localization pattern of classical *WOX* proteins, such as *AtWOX5* [26]. In contrast, the dual (nuclear and plasma membrane) localization of *SmWOX15* is relatively uncommon among *WOX* family members. Structural prediction indicates that *SmWOX15* (837 amino acids) is substantially longer than *SmWOX5* (163 amino acids) and harbors numerous putative phosphorylation sites particularly enriched in its N-terminal region (residues 1–300). Previous studies have shown that the subcellular distribution of transcription factors can be dynamically regulated by post-translational modifications, including phosphorylation [34,35], and certain transcription factors undergo transient retention at the plasma membrane via interactions with membrane-anchored proteins [36]. Based on these observations, we hypothesize that the plasma membrane association of *SmWOX15* may represent a dynamic “signal-sensing” state. It is tempting to speculate that, upon perception of specific extracellular or intracellular cues, *SmWOX15* could be phosphorylated and subsequently translocated into the nucleus to modulate transcriptional programs. However, we acknowledge that this model is currently speculative and requires direct experimental validation, such as phosphorylation assays, time-course imaging, and stimulus-response experiments, in future studies. By comparison, *SmWOX5* possesses a more compact structure and a highly specialized functional role; its exclusive nuclear localization reflects the evolutionary conservation of its function in RAM maintenance.

In summary, *SmWOX5* and *SmWOX15* likely exhibit functional divergence during root development and adventitious root formation in pepino: *SmWOX5* appears to be primarily dedicated to sustaining the root apical meristem, whereas *SmWOX15* may act as a regulatory node integrating environmental or developmental signals to modulate adventitious root initiation. However, the functional roles of *SmWOX5* and *SmWOX15* in root development and cutting propagation need to be further validated through stable genetic transformation and ectopic expression studies. Future work will focus on generating transgenic pepino lines overexpressing these two genes to elucidate their common and specific functions, which will provide valuable genetic resources for pepino breeding programs.

## 5. Conclusions

In this study, a total of 15 *WOX* family genes were identified in the pepino (*Solanum muricatum*) genome. Phylogenetic analysis grouped these genes into three evolutionarily distinct clades: the WUS clade (11 members), the Intermediate clade (3 members), and the Ancient clade (1 member). Tissue-specific expression profiling revealed that *SmWOX5* and *SmWOX15* exhibited markedly elevated expression levels in roots. Subcellular localization assays demonstrated that *SmWOX5* is exclusively localized to the nucleus, whereas *SmWOX15* displays predominant nuclear localization with partial accumulation at the plasma membrane. Collectively, these findings identify *SmWOX5* and *SmWOX15* as promising candidate genes and provide a theoretical foundation for enhancing adventitious root formation in pepino cuttings, and thereby improving cutting propagation efficiency, through targeted regulation of these two genes.

## Figures and Tables

**Table 1:** WOX gene family identified in the pepino genome.

Gene ID	Gene	Chromosome	Gene Location	
			Start	End
Smu01G013420.1	*SmWOX1*	chr01	49000495	49003949
Smu02G019510.1	*SmWOX2*	chr02	60672693	60676128
Smu02G024710.1	*SmWOX3*	chr02	65317825	65320857
Smu02G026070.1	*SmWOX4*	chr02	66240598	66242021
Smu03G012910.1	*SmWOX5*	chr03	63539927	63540501
Smu03G029870.1	*SmWOX6*	chr03	79541167	79544057
Smu06G027800.1	*SmWOX7*	chr06	71885779	71888578
Smu06G030420.1	*SmWOX8*	chr06	74005127	74006349
Smu06G030780.1	*SmWOX9*	chr06	74230027	74231717
Smu08G014760.1	*SmWOX10*	chr08	70791392	70798010
Smu11G004630.1	*SmWOX11*	chr11	3584253	3585690
Smu11G022710.1	*SmWOX12*	chr11	84825209	84827440
Smu11G022720.1	*SmWOX13*	chr11	84850796	84852206
Smu11G025670.1	*SmWOX14*	chr11	87952453	87959022
Smu12G017550.1	*SmWOX15*	chr12	96029146	96038223

## Data Availability

The CDS sequences of SmWOX5 and SmWOX15 have been deposited in the GenBase database of the China National Center for Bioinformation (CNCB) under accession numbers C_AA491322.1 (*SmWOX5*) and C_AA491323.1 (*SmWOX15*).

## Supplementary Materials

The following supporting information can be downloaded at: https://www.mdpi.com/article/10.3390/cimb48080782/s1.
