# Supplementary material for: Genome-Wide Identification and Expression Analysis of the WOX Gene Family in Pepino (Solanum muricatum), Followed by Cloning and Subcellular Localization of SmWOX5 and SmWOX15
Source: Curr Issues Mol Biol. 2026 Jul 31;48(8):782. doi: 10.3390/cimb48080782 (PMC13511356; doi:10.3390/cimb48080782)
Supplement: Supplementary file 1 [file cimb-48-00782-s001.zip › cimb-4439603-supplementary.pdf]

A. The qRT-PCR reaction system for SmWOX gene in pepino (Solanum muricatum).

Composition	Volume
2×FastReal qPCR PreMix	10μL
Forward primer（10μM）	0.6μL
Reverse primer（10μM）	0.6μL
cDNA（100ng/μL）	1.5μL
RNase-Free ddH2O	7.3μL
Total	20μL

Cycling program:

Temperature	Time	Cycle times
95℃	2min	
95℃	5s	40 cycles
60℃	15s	

B. The PCR reaction system for cloning SmWOX5 and SmWOX15

Composition	Volume
Primer STAR Max Premix（2×）	10μL
Forward primer（10μM）	1μL
Reverse primer（10μM）	1μL
cDNA（100ng/μL）	2μL
RNase-Free ddH2O	6μL
Total	20μL

Thermal cycling program:

Temperature	Time	Cycle times
95℃	3min	
98℃	10s	32 cycles
55℃	15s	
72℃	2min	
72℃	5min	

Figure S1. PCR amplification system and thermal cycling protocol used in this study.

A. Agarose gel electrophoresis of PCR-amplified SmWOX5 and SmWOX15 fragments.

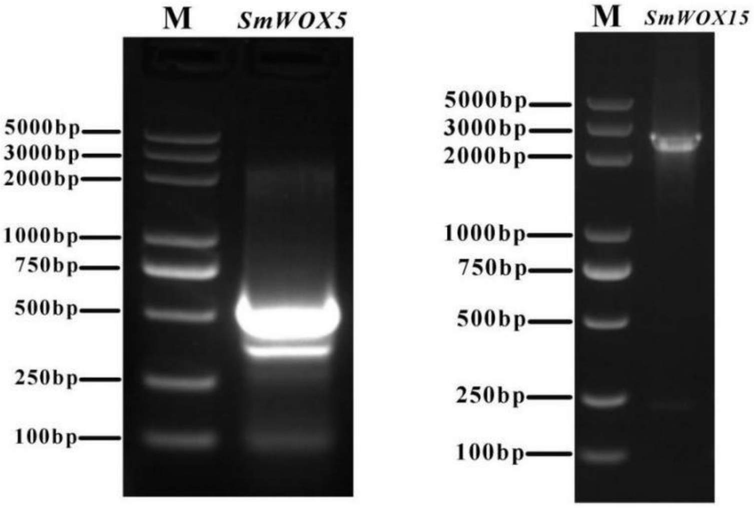

B.Colony PCR identification of recombinant pCAMBIA2300-SmWOX5-GFP and pCAMBIA2300-SmWOX15-GFP.

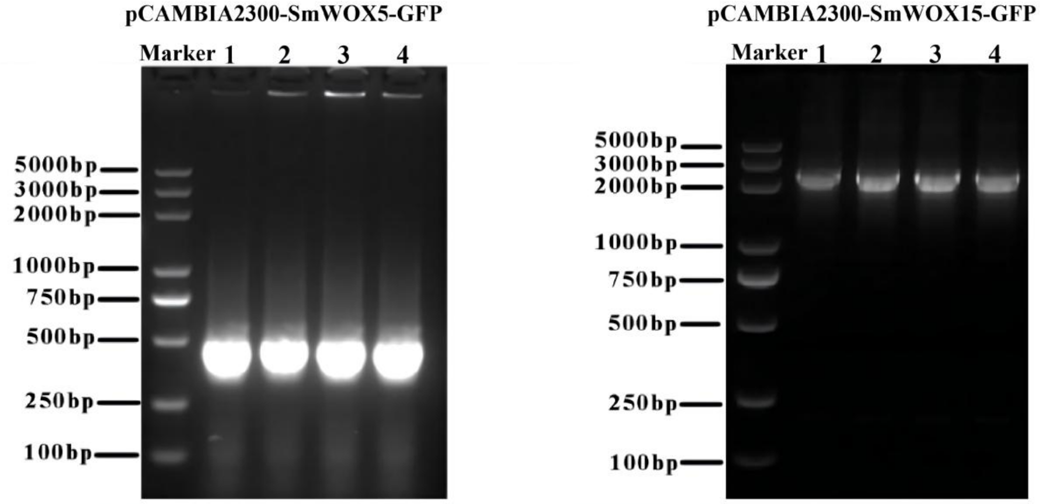

Figure S2. Agarose gel electrophoresis of PCR-amplified fragments and colony PCR verification of recombinant vectors.

A. Transmembrane domain analysis of SmWOX5 and SmWOX15.

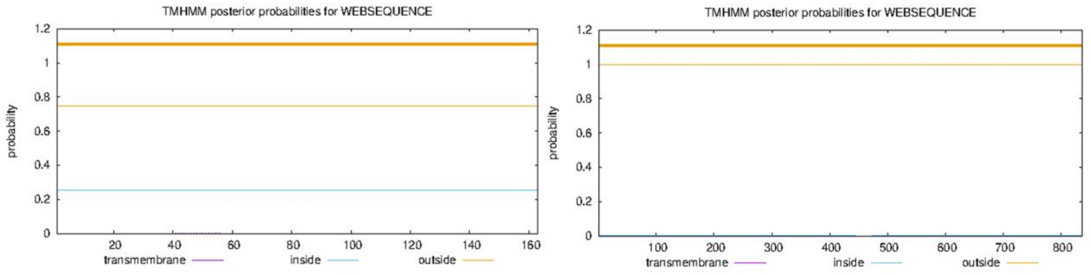

SmWOX5 **SmWOX15**

B. Signal peptide prediction analysis of SmWOX5 and SmWOX15.

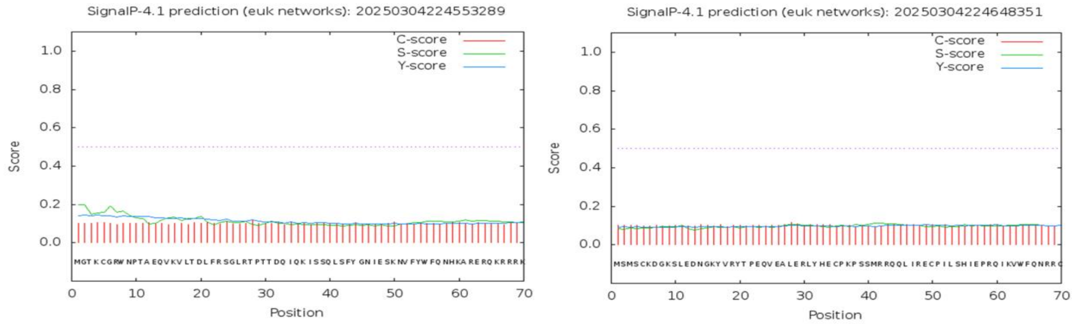

SmWOX5 **SmWOX15**

C. Prediction of protein phosphorylation of SmWOX5 and SmWOX15.

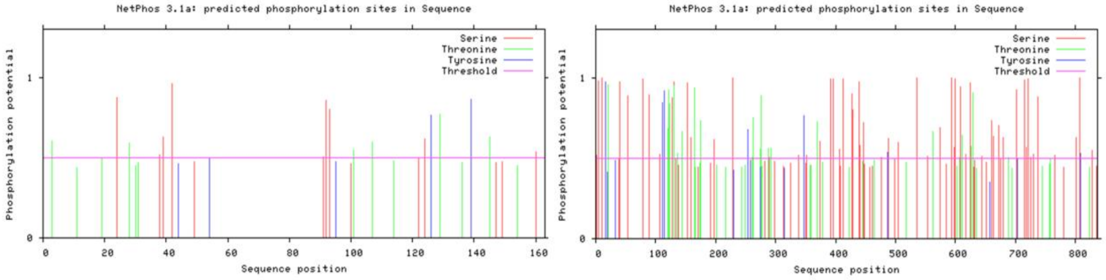

SmWOX5 **SmWOX15**

D. Protein structure prediction of SmWOX5 and SmWOX15.

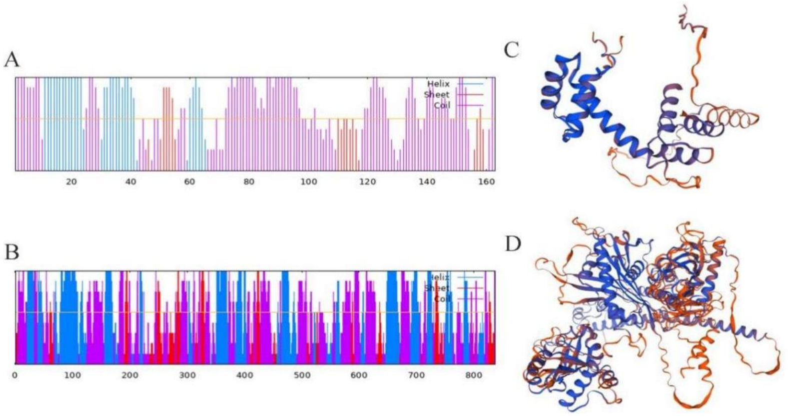

(A) SmWOX5 secondary structure; (B) SmWOX15 secondary structure; (C) SmWOX5 tertiary structure; (D) SmWOX15 tertiary structure

Figure S3. Bioinformatics analysis of *SmWOX5* and *SmWOX15*.

Table S1 PCR primer sequences used in this study.

A.Primers used for qRT-PCR analysis of *SmWOX* genes in different tissues.

Gene	Forward primer（5'-3'）	Reverse primer（5'-3'）
UBI119	TCGTAAGGAGTGCCCTAATGCTGA	CAATCGCCTCCAGCCTTGTTGTAA
SmWOX1	GTAGTGGAGGTGGAGAACAATCAAC	AAGAAGAAGAGGAGGACGAGGAAC
SmWOX2	TGGTGAAGATGCGGTGCTTATTC	TGCTGGAGTGGCTGAAGAGTG
SmWOX3	CAGAGGTGGAGACAGAGGTTGAG	CCATTGAAGTAGGCATGTGAGAAGAC
SmWOX4	TTGAGACAGTACGGAAAGATTGAAG	GCAGAGGCAGCAGCAATGAG
SmWOX5	TGGAATCCTACGGCGGAACAG	CAGTGGTCGGAGTACGGAGTC
SmWOX6	GGAGAACAAAGAAGGAGCCTATTAG	TGATGGTGGTGGGTGGTGAAC
SmWOX7	GCCAGCCTTAGTAATGAAGAACCATC	CTCCTCCAAGTGAAGACGAAGAAAC
SmWOX8	TCCAAAACCACAAAGCTAGAGACAG	GAGGTGAAGGAGGAGGAGGAATAG
SmWOX9	TACAGTTTCTTCTTCATCGTCGTCTAC	TTGAAGGACAAGTACCAGTAGTAACAC
SmWOX10	GTGGAGTGTGCCTGAAGTGTTG	CGCCGCCATAGTAGTCCTCTG
SmWOX11	GCTGCTGCTCCTTCACTTACAC	GTGGTTGTGGTATCATTATTGGTTGTG
SmWOX12	CACCCTCACTCTCCTCCCATATTC	TTGAGTTGTTGTTGATGATGATGTGTC
SmWOX13	ACAAACACAAATACCCTCTTTCATTG	ATGCTTCTTTGATTCCACCCTCTTG
SmWOX14	ACGATGGCTGGTCAATGTTAAGTTC	GAGGAGGTGGCGAGGTTCTTC
SmWOX15	TCCTCGTGACTTCTGGCTGTTAC	TTCTGAACTTGCGGCATACTTGG

B. Primers used for cloning of *SmWOX5* and *SmWOX15*.

Primer names	Primer sequence（5'-3'）
SmWOX5-F	ATGGGAACAAAATGTGGTCGATG
SmWOX5-R	TGAGAAGAAGCTTAGACGCAAATC
SmWOX15-F	ATGTCAATGTCCTGCAAGGATGGTA
SmWOX15-R	AAACAAACGACCAATTCAC

C. Primers used for subcellular localization vector construction.

Primer names	Primer sequence（5'-3'）
SmWOX5-2300-F	AGAACACGGGGGACGAGCTCATGGGAACAAAATGTGGTCGATG
SmWOX5-2300-R	ACCATGGTGTCGACTCTAGATGAGAAGAAGCTTAGACGCAAATC
SmWOX15-2300-F	AGAACACGGGGGACGAGCTCATGTCAATGTCCTGCAAGGATGGTA
SmWOX15-2300-R	ACCATGGTGTCGACTCTAGA AAACAAACGACCAATTCAC

Table S2. Physicochemical properties of the WOX family members in pepino.

Protein	Number of Amino Acid	Molecular Weight/Da	Isoelectric points	Instability Index	Aliphatic Index	GRAVY
SmWOX1	271	29872.46	5.49	68.53	72.75	-0.404
SmWOX2	357	39761.27	7.26	62.44	61.99	-0.634
SmWOX3	272	30533.12	5.53	53.96	54.85	-0.859
SmWOX4	297	33800.98	6.26	61.34	52.26	-1.031
SmWOX5	163	19263.73	5.96	69.53	68.77	-0.696
SmWOX6	392	43951.64	7.22	56.36	54.16	-0.858
SmWOX7	228	25026.72	5.47	66.43	67.02	-0.493
SmWOX8	172	20020.13	9.98	52.07	49.3	-0.855
SmWOX9	240	26738.99	8.73	62.34	63	-0.541
SmWOX10	898	98884.23	5.63	54.29	87.03	-0.103
SmWOX11	239	27250.5	8.37	61.15	56.32	-0.977
SmWOX12	202	23292.73	9.43	42.86	64.65	-0.752
SmWOX13	218	25087.67	9.32	40.93	66.7	-0.734
SmWOX14	841	92081.04	5.84	51.56	86.88	-0.107
SmWOX15	837	92283.76	6.11	47.07	86.69	-0.146

Table S3 Relative expression levels (mean ± SD) of *SmWOX5* and *SmWOX15* in pepino roots.

Gene	Replicate 1	Replicate 2	Replicate 3	Mean ± SD
SmWOX5	0.543	0.325	0.518	0.462 ± 0.119
SmWOX15	1.021	0.595	0.979	0.865 ± 0.237

Note: Values represent relative expression calculated using the 2−ΔΔCT method, with leaf tissue as the calibrator (2−ΔΔCT=1). Data are shown as mean ± SD (n=3 biological replicates). Statistical significance: Student's t-test, p=0.047.
